# Supplementary material for: Experimental verification of multipartite entanglement in quantum networks
Source: Nat Commun. 2016 Nov 9;7:13251. doi: 10.1038/ncomms13251 (PMC5105160; doi:10.1038/ncomms13251)
Supplement: Supplementary Information — Supplementary Figures 1-2, Supplementary Notes 1-2 and Supplementary References. [file ncomms13251-s1.pdf]

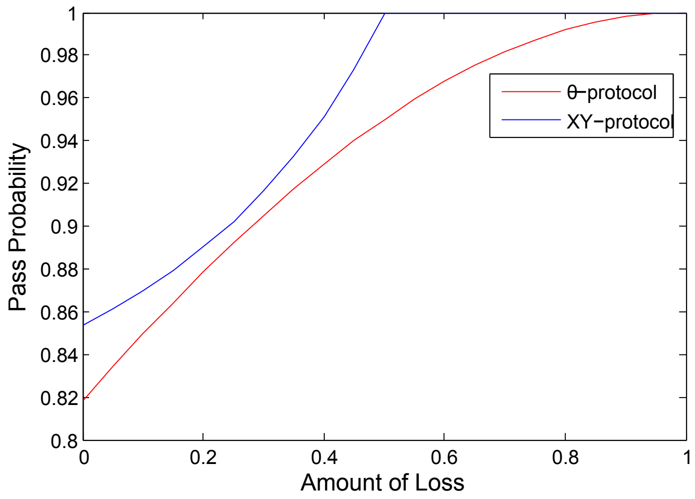

Supplementary Figure 1 – Loss tolerance of the original and new verification protocols. The θ-protocol test performs better than the XY-protocol test and is still viable after 50% loss.

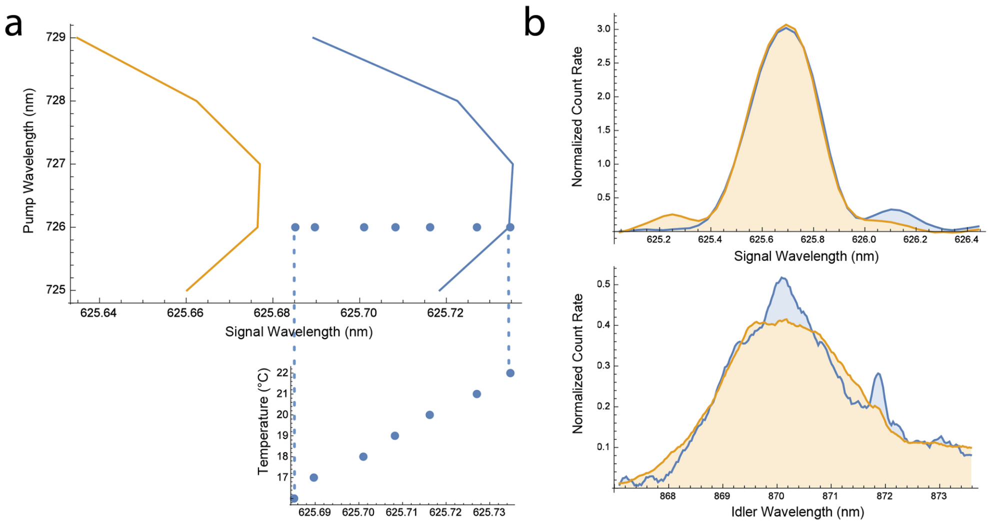

Supplementary Figure 2 – **Tuning the photonic crystal fibre sources.** Spectra of the microstructured photonic crystal fiber sources. **(a)** Central wavelengths for source 1 (orange line) and source 2 (blue line) with varying pump wavelength. The temperature tuning of source 2 (blue points) is also shown. **(b)** Spectra of signal photons (top) and idler photons (bottom) from source 1 (orange) and source 2 (blue) tuned at $23.7°C$ with a 726 nm pump.

Supplementary Note 1

In this section we provide further details of the θ-protocol presented in the main text (see Fig. 1). Note that the proofs for the XY-protocol follow easily as a special case with only two measurement settings.

Correctness of the θ-protocol

Here we prove that the n-qubit GHZ state passes the verification test with probability 1. The measurements that the parties perform in the X-Y plane are equivalent to rotation operators around the Z axis of the Bloch sphere:

$$R_z(\theta_j) = \begin{bmatrix} 1 & 0 \\ 0 & e^{-i\theta_j} \end{bmatrix} \tag{1}$$

followed by a measurement in the Pauli X basis. After the application of the rotation operators $R_z(\theta_j)$ on an n-qubit GHZ state, we end up with the state $\frac{1}{\sqrt{2}}\big(|0\rangle^{\otimes n} + e^{-i\Theta} |1\rangle^{\otimes n} \big)$, where $\Theta = \sum_{j=1}^{n} \theta_j$. When $\Theta = 0 \pmod{2\pi}$ the shared state written in the Pauli X basis is given by a linear summation of terms with an even number of $|-\rangle$ states for the parties. On the other hand when $\Theta = \pi \pmod{2\pi}$ the shared state is given by a linear summation of terms with an odd number of $|-\rangle$ states for the parties. Thus, when the parties measure their qubits in the Pauli X basis the parity of their measurements will be zero if $\Theta = 0 \pmod{2\pi}$ and one if $\Theta = \pi \pmod{2\pi}$. In other words, the test will always be passed with unit probability.

Security in the Honest Model

Now we prove a lower bound for the fidelity of the shared state, when all parties are honest, that depends on the pass probability of the test $P(\rho)$. We will specifically prove the following theorem:

Theorem 1 (Honest Case)**.** *Let ρ be the state shared between n parties. If $F(\rho, |G_0^n\rangle) := \langle G_0^n| \rho |G_0^n\rangle$, where $|G_0^n\rangle$ is an n-qubit GHZ state, then $F(\rho) \geq 2P(\rho) - 1$.*

Let us define a test in order to verify a 'rotated' GHZ state, namely $|G_\Theta^n\rangle = 1/\sqrt{2}(|0\rangle^{\otimes n} + e^{-i\Theta} |1\rangle^{\otimes n})$, where $\Theta \in [0, 2\pi)$. Here, the sum of the angles of the parties has to comply with the condition: $\sum_{j=1}^{n} \theta_j - \Theta \equiv 0 \pmod{\pi}$. The test that we are interested in is the following:

$$\bigoplus_{j=1}^{n} Y_j = \frac{\sum_{j=1}^{n} \theta_j - \Theta}{\pi} \pmod{2} \tag{2}$$

Let $\{P^n_\Theta, I - P^n_\Theta\}$ be the POVM that corresponds to the above test. We will prove by induction that:

$$P^n_\Theta = |G^n_\Theta\rangle\langle G^n_\Theta| + \frac{1}{2} I^\Theta_n \tag{3}$$

where I^Θ_n is the projection on the space orthonormal to $|G^n_\Theta\rangle$ and $\left|G^n_{\Theta+\pi}\right\rangle$.

For $n = 1$ we have that $P^1_\Theta = |G^1_{\theta_1}\rangle\langle G^1_{\theta_1}|$ so the statement holds. We assume it is true for n and we show the statement for $n+1$.

Let $\{P^{n+1}_\Theta(\theta_1), I - P^{n+1}_\Theta(\theta_1)\}$ be the POVM that corresponds to the test for a given angle θ_1. There are two cases:

1. Party 1 outputs $Y_1 = 0$. Then, the following equality should hold:
$$\bigoplus_{j=2}^{n+1} Y_j = \frac{\sum_{j=2}^{n+1} \theta_j - (\Theta - \theta_1)}{\pi} \pmod 2 \tag{4}$$
2. Party 1 outputs $Y_1 = 1$. Then, the following equality should hold:
$$\bigoplus_{j=2}^{n+1} Y_j = \frac{\sum_{j=2}^{n+1} \theta_j - (\Theta - \theta_1 + \pi)}{\pi} \pmod 2 \tag{5}$$

Let $\Theta' \equiv \Theta - \theta_1 \pmod{2\pi}$. It is evident that the first outcome of the test is equivalent to $P^n_{\Theta'}$ and the second to $I - P^n_{\Theta'}$. For any given θ_1, we have:

$$\begin{aligned} P^{n+1}_\Theta(\theta_1) &= |G^1_{\theta_1}\rangle\langle G^1_{\theta_1}| \otimes P^n_{\Theta'} + \left|G^1_{\theta_1+\pi}\right\rangle\left\langle G^1_{\theta_1+\pi}\right| \otimes (I - P^n_{\Theta'}) \\ &= |G^1_{\theta_1}\rangle\langle G^1_{\theta_1}| \otimes |G^n_{\Theta'}\rangle\langle G^n_{\Theta'}| + \left|G^1_{\theta_1+\pi}\right\rangle\left\langle G^1_{\theta_1+\pi}\right| \otimes \left|G^n_{\Theta'+\pi}\right\rangle\left\langle G^n_{\Theta'+\pi}\right| \\ &+ \frac{1}{2}\Big(|G^1_{\theta_1}\rangle\langle G^1_{\theta_1}| + \left|G^1_{\theta_1+\pi}\right\rangle\left\langle G^1_{\theta_1+\pi}\right|\Big) \otimes I^{\Theta'}_n \\ &= |G^{n+1}_\Theta\rangle\langle G^{n+1}_\Theta| + |\Phi_{\theta_1}\rangle\langle\Phi_{\theta_1}| + \frac{1}{2} I_1 \otimes I^{\Theta'}_n \end{aligned} \tag{6}$$

where we define:

$$|\Phi_a\rangle = \frac{1}{\sqrt{2}}\big(\left|G^1_a\right\rangle\left|G^n_{\Theta-a}\right\rangle - \left|G^1_{a+\pi}\right\rangle\left|G^n_{\Theta-a+\pi}\right\rangle\big) \tag{7}$$

It is straightforward to verify that:

$$I^\Theta_{n+1} = |\Phi_{\theta_1}\rangle\langle\Phi_{\theta_1}| + |\Phi_{\theta_1+\frac{\pi}{2}}\rangle\langle\Phi_{\theta_1+\frac{\pi}{2}}| + I_1 \otimes I^{\Theta'}_n \tag{8}$$

where as before I^Θ_{n+1} is the projection on the space orthonormal to $\left|G^{n+1}_\Theta\right\rangle$ and $\left|G^{n+1}_{\Theta+\pi}\right\rangle$. Since angle θ_1 is chosen uniformly at random in $[0, \pi)$, we have that:

$$\begin{aligned} P^{n+1}_\Theta &= \frac{1}{\pi}\int_0^\pi P^{n+1}_\Theta(\theta_1) d\theta_1 \\ &= \frac{1}{\pi}\int_0^{\pi/2} \Big[P^{n+1}_\Theta(\theta_1) + P^{n+1}_\Theta(\theta_1 + \frac{\pi}{2})\Big] d\theta_1 \\ &= |G^{n+1}_\Theta\rangle\langle G^{n+1}_\Theta| + \frac{1}{2} I^\Theta_{n+1} \end{aligned} \tag{9}$$

For $\Theta = 0 \pmod{2\pi}$, we can easily infer the basic argument of the proof, that the test is equivalent to performing the POVM $\{P_0^n, I - P_0^n\}$. We can therefore express any state ρ with fidelity $F(\rho)$ to the GHZ state as $\rho = F(\rho)|G_0^n\rangle\langle G_0^n| + (1 - F(\rho))\chi$, where χ is a $2^n \times 2^n$ density matrix with zero in the place of $|G_0^n\rangle\langle G_0^n|$. We then have $P(\rho) = \mathrm{Tr}(P_0^n \rho) \leq \frac{1}{2} + \frac{F(\rho)}{2}$.

Security in the Dishonest Model

Figures of merit for the dishonest case. Without loss of generality, the source generates a state $\sum_r p_r |r\rangle\langle r| \otimes |\Psi_r\rangle\langle\Psi_r|_{\mathcal{HDE}}$ where r corresponds to some classical information controlled by the dishonest players, and $\mathcal{HDE}$ are respectively the Hilbert space of the honest parties, the dishonest parties and the external environment, which no parties can control.

Here we prove a lower bound for the fidelity of the shared state, when the $n-k$ parties are dishonest and there are no loss in the system. Since we consider that the dishonest parties can collaborate between themselves and with the source, any security statement should consider that they can apply any operation U_r (possibly depending on r) to their part of the state that works to their advantage. More specifically, we prove the following theorem:

Theorem 2 (Dishonest Case)**.** *Let $\rho = \sum_{r=1}^{R} p_r |r\rangle\langle r| \otimes \rho_r$ be the state shared between n parties in the space $\mathcal{HD}$. If $F'(\rho) := \sum_r p_r \max_{U_{n-k}^r} F\big((\mathbb{I}_k \otimes U_{n-k}^r)\rho_r(\mathbb{I}_k \otimes (U_{n-k}^r)^\dagger), |G_0^n\rangle\big)$, where U_{n-k}^r are operators on the space of the dishonest parties, then $F'(\rho) \geq 4P(\rho) - 3$.*

Proof.

Case 1 : Pure state. We first consider the case without classical information r and without environment, *i.e.* where ρ is a pure state $|\Psi\rangle\langle\Psi|_{\mathcal{HD}}$. We write

$$|\Psi\rangle = \left|G_\theta^k\right\rangle|\Psi_\theta\rangle + \left|G_{\pi+\theta}^k\right\rangle|\Psi_{\pi+\theta}\rangle + |\mathcal{X}\rangle \tag{10}$$

where $\theta = \sum_{j\in H}\theta_j \pmod{\pi}$ is the honest angle, H is the set of the honest parties and $\left|G_\alpha^k\right\rangle = 1/\sqrt{2}(|0\rangle^{\otimes k} + e^{i\alpha}|1\rangle^{\otimes k})$ for any angle α. Note that the component of the honest parties in $|\mathcal{X}\rangle$ is orthogonal to both $\left|G_\theta^k\right\rangle$ and $\left|G_{\pi+\theta}^k\right\rangle$.

The dishonest parties want to know in which of the two states $\left|G_\theta^k\right\rangle$ and $\left|G_{\theta+\pi}^k\right\rangle$ the state the honest parties share will collapse into after the measurement, and by consequence what will be the honest output $Y_H = \bigoplus_{i\in H} Y_i$. They will perform

a Helstrom measurement on their share in order to distinguish between $|\Psi_\theta\rangle$ and $|\Psi_{\theta+\pi}\rangle$. This measurement is optimal and gives the following bound:

$$\Pr[\text{guess } Y_H|\theta] = \frac{1}{2} + \frac{1}{2}\Big\| |\Psi_\theta\rangle\langle\Psi_\theta| - |\Psi_{\theta+\pi}\rangle\langle\Psi_{\theta+\pi}| \Big\| \tag{11}$$

To calculate the above norm, we make use of a known property, that the trace norm of a Hermitian matrix is equal to the sum of the absolute values of its eigenvalues. After some simple calculations we can verify that the above probability is equal to:

$$\begin{aligned}\Pr[\text{guess } Y_H|\theta] &= \frac{1}{2} + \frac{1}{2}\sqrt{\big(\||\Psi_\theta\rangle\|^2 + \||\Psi_{\theta+\pi}\rangle\|^2\big)^2 - 4|\langle\Psi_\theta|\Psi_{\theta+\pi}\rangle|^2} \\ &\leq \frac{1}{2} + \frac{1}{2}\Big(\frac{\big(\||\Psi_\theta\rangle\|^2 + \||\Psi_{\theta+\pi}\rangle\|^2\big)^2 - 4|\langle\Psi_\theta|\Psi_{\theta+\pi}\rangle|^2 + 1}{2}\Big) \\ &= \frac{3}{4} + \frac{1}{4}\Big(\big(\||\Psi_\theta\rangle\|^2 + \||\Psi_{\theta+\pi}\rangle\|^2\big)^2 - 4|\langle\Psi_\theta|\Psi_{\theta+\pi}\rangle|^2\Big)\end{aligned} \tag{12}$$

We now perform a Schmidt decomposition of $\left|G^k_\theta\right\rangle|\Psi_\theta\rangle + \left|G^k_{\theta+\pi}\right\rangle|\Psi_{\theta+\pi}\rangle$:

$$\left|G^k_\theta\right\rangle|\Psi_\theta\rangle + \left|G^k_{\theta+\pi}\right\rangle|\Psi_{\theta+\pi}\rangle = \left|A^0_\theta\right\rangle\left|B^0_\theta\right\rangle + \left|A^1_\theta\right\rangle\left|B^1_\theta\right\rangle \tag{13}$$

where $\langle A^0_\theta|A^1_\theta\rangle = \langle B^0_\theta|B^1_\theta\rangle = 0$. We use the following normalization: $\| |A^0_\theta\rangle \|^2 = \| |A^1_\theta\rangle \|^2 = 1$, $\| |B^0_\theta\rangle \|^2 = p_\theta$, $\| |B^1_\theta\rangle \|^2 = q_\theta$. There exist $z_0, z_1 \in \mathbb{C}$ such that:

$$\left|A^0_\theta\right\rangle = z_0\left|G^k_\theta\right\rangle + z_1\left|G^k_{\theta+\pi}\right\rangle \quad \text{and} \quad \left|A^1_\theta\right\rangle = z_1^*\left|G^k_\theta\right\rangle - z_0^*\left|G^k_{\theta+\pi}\right\rangle \tag{14}$$

where $|z_0|^2 + |z_1|^2 = 1$, which gives us:

$$\begin{aligned}\left|A^0_\theta\right\rangle\left|B^0_\theta\right\rangle + \left|A^1_\theta\right\rangle\left|B^1_\theta\right\rangle &= (z_0\left|G^k_\theta\right\rangle + z_1\left|G^k_{\theta+\pi}\right\rangle)|B_0\rangle + (z_1^*\left|G^k_\theta\right\rangle - z_0^*\left|G^k_{\theta+\pi}\right\rangle)|B_1\rangle \\ &= \left|G^k_\theta\right\rangle(z_0\left|B^0_\theta\right\rangle + z_1^*\left|B^1_\theta\right\rangle) + \left|G^k_{\theta+\pi}\right\rangle(z_1\left|B^0_\theta\right\rangle - z_0^*\left|B^1_\theta\right\rangle)\end{aligned} \tag{15}$$

and from Eq. (13) we have:

$$\begin{aligned}|\Psi_\theta\rangle &= z_0\left|B^0_\theta\right\rangle + z_1^*\left|B^1_\theta\right\rangle \\ |\Psi_{\theta+\pi}\rangle &= z_1\left|B^0_\theta\right\rangle - z_0^*\left|B^1_\theta\right\rangle\end{aligned} \tag{16}$$

Since $|A^0_\theta\rangle$ and $|A^1_\theta\rangle$ are on the same subspace as $\left|G^k_\theta\right\rangle$ and $\left|G^k_{\theta+\pi}\right\rangle$, there exist $x \in \mathbb{R}, y \in \mathbb{C}$ such that:

$$\left|A^0_\theta\right\rangle = x\left|0^k\right\rangle + y\left|1^k\right\rangle \quad \text{and} \quad \left|A^1_\theta\right\rangle = y^*\left|0^k\right\rangle - x\left|1^k\right\rangle \tag{17}$$

where $x^2 + |y|^2 = 1$ (we can assume that $x \in \mathbb{R}$ up to a global phase on $|A_0\rangle$ and $|A_1\rangle$). Then:

$$|z_0|^2 = |\langle A^0_\theta|G^\theta_k\rangle|^2 = \frac{1}{2}|x + ye^{i\theta}|^2 \tag{18}$$

and since $y \in \mathcal{C}$, we rewrite $y = |y|e^{i\alpha}$ and get:

$$|z_0|^2 = \frac{1}{2}\left|x + |y|e^{i\theta+\alpha}\right|^2 = \frac{1}{2}(1 + 2x|y|\cos(\theta+\alpha)) \tag{19}$$

Using $|z_0|^2 + |z_1|^2 = 1$, we have $|z_1|^2 = \frac{1}{2}(1 - 2x|y|\cos(\theta+\alpha))$. Also, from $x^2, |y|^2 \geq 0$ and $x^2 + |y|^2 = 1$, we have that $x^2|y|^2 \leq 1/4$. This gives us:

$$\begin{aligned} |\langle\Psi_\theta|\Psi_{\theta+\pi}\rangle|^2 &= (p_\theta - q_\theta)^2|z_0|^2|z_1|^2 = (p_\theta - q_\theta)^2\frac{1}{4}(1 - 4x^2|y|^2\cos^2(\theta+\alpha)) \\ &\geq (p_\theta - q_\theta)^2\frac{1}{4}(1 - \cos^2(\theta+\alpha)) = (p_\theta - q_\theta)^2\frac{1}{4}\sin^2(\theta+\alpha) \end{aligned} \tag{20}$$

We then revisit Eq. (12):

$$\Pr[\text{guess } Y_H|\theta] \leq \frac{3}{4} + \frac{1}{4}\big((p_\theta + q_\theta)^2 - (p_\theta - q_\theta)^2\sin^2(\theta+\alpha)\big) \tag{21}$$

Now, let us consider the optimal local operation that the dishonest parties can perform on their state, in order to maximize their cheating probability. If the reduced density matrices of the honest parties of the ideal state $|G_0^n\rangle$ and the state ρ are σ_H and ρ_H respectively, it holds that there exists a local operation R on the dishonest state that maximizes the fidelity:

$$F'(\rho) = F((I \otimes R)\,|\Psi\rangle\,, |G_0^n\rangle) = F(\sigma_H, \rho_H) \tag{22}$$

Let us decompose $|G_0^n\rangle$ in the same orthonormal bases for the honest parties, as we did for $|\Psi\rangle$. We have $|G_0^n\rangle = |A_\theta^0\rangle\,|C^0\rangle + |A_\theta^1\rangle\,|C^1\rangle$. Then:

$$\sigma_H = \frac{1}{2}\big(|A_\theta^0\rangle\langle A_\theta^0| + |A_\theta^1\rangle\langle A_\theta^1|\big) \tag{23}$$

$$\rho_H = p_\theta|A_\theta^0\rangle\langle A_\theta^0| + q_\theta|A_\theta^1\rangle\langle A_\theta^1| + \mathrm{Tr}_{n-k}|\mathcal{X}\rangle\,\langle\mathcal{X}| \tag{24}$$

and we can express fidelity $F'(\rho) = \mathrm{Tr}[\sqrt{\sqrt{\rho_H}\sigma_H\sqrt{\rho_H}}]^2$, which gives:

$$\begin{aligned} F'(\rho) &= \frac{1}{2}(\sqrt{p_\theta} + \sqrt{q_\theta})^2 = \frac{p_\theta + q_\theta}{2} + \sqrt{p_\theta q_\theta} \\ &\geq \frac{(p_\theta + q_\theta)^2}{2} + 2p_\theta q_\theta = (p_\theta + q_\theta)^2 - \frac{(p_\theta - q_\theta)^2}{2} \end{aligned} \tag{25}$$

because for all non-negative p and q such that $p + q \leq 1$, it holds that $p + q \geq (p+q)^2$ for $p + q \leq 1$ and also that $\sqrt{pq} \geq 2pq$. Let us note here that whatever decomposition we do to the state $|\Psi\rangle$, the sum $(p_\theta + q_\theta)$ is a constant that always equals $\|\,|\Psi_\theta\rangle\,\|^2 + \|\,|\Psi_{\theta+\pi}\rangle\,\|^2$. It follows that $(p_\theta - q_\theta)^2$ is lower bounded by the

constant $2((p_\theta+q_\theta)^2 - F'(\rho))$. Since θ is chosen uniformly at random, we have that:

$$P(\rho) = \frac{1}{\pi}\int_0^\pi \Pr[\text{guess } Y_H|\theta] \tag{26}$$

$$\leq \frac{3}{4} + \frac{1}{4}\Big((p_\theta+q_\theta)^2 - \frac{1}{\pi}\int_0^\pi (p_\theta - q_\theta)^2 \sin^2(\theta+\alpha) d\theta\Big) \tag{27}$$

$$\leq \frac{3}{4} + \frac{1}{4}\Big((p_\theta+q_\theta)^2 + F'(\rho) - (p_\theta+q_\theta)^2\Big) \tag{28}$$

$$\leq \frac{3}{4} + \frac{1}{4}F'(\rho) \tag{29}$$

Case 2 : No classical information, mixed state. We consider the case where $\rho = \sum_j q_j |\Psi_j\rangle\langle\Psi_j|_{\mathcal{HD}}$. Since the two functions $P(\cdot)$ and $F(\cdot)$ are linear, we can write

$$P(\rho) = \sum_j q_j P(|\Psi_j\rangle\langle\Psi_j| \leq \frac{3}{4} + \frac{1}{4}\sum_j q_j F'(|\Psi_j\rangle\langle\Psi_j|) = \frac{3}{4} + \frac{1}{4}F'(\rho) \tag{30}$$

Case 3 : General Case. We write $\rho = \sum_{r=1}^R p_r |r\rangle\langle r| \otimes \rho_r$. We then write

$$P(\rho) = \sum_r p_r P(\rho_r) = \frac{3}{4} + \frac{1}{4}\sum_r p_r(F(\rho_r)) \tag{31}$$

$$= \frac{3}{4} + \frac{1}{4}\sum_r p_r \max_{U^r_{n-k}} F\big((\mathbb{I}_k \otimes U^r_{n-k})\rho_r(\mathbb{I}_k \otimes (U^r_{n-k})^\dagger), |G^n_0\rangle\big) \tag{32}$$

□

Corollary 1. *Let ρ be the state shared between n parties. If $F'(\rho) := \max_U F\big((\mathbb{I}_k \otimes U_{n-k})\rho(\mathbb{I}_k \otimes U_{n-k}), |G^n_0\rangle\big) = \frac{1}{2}$, where U is an operator on the space of the dishonest parties, then*

1. *if the parties run the θ-protocol, $P(\rho|\theta\text{-protocol}) \leq \frac{1}{2} + \frac{1}{\pi}$.*
2. *if the parties run the XY-protocol, $P(\rho|XY\text{-protocol}) \leq \cos^2(\frac{\pi}{8})$.*

Proof. We will first show the upper bound of the pass probability for the θ-protocol and then examine the special case where the honest angle θ is either equal to 0 or $\pi/2$. Following the derivations of Eq. (12) and Eq. (21) we have

$$\Pr[\text{guess } Y_H|\theta] = \frac{1}{2} + \frac{1}{2}\sqrt{\big(|||\Psi_\theta\rangle||^2 + |||\Psi_{\theta+\pi}\rangle||^2\big)^2 - 4|\langle\Psi_\theta|\Psi_{\theta+\pi}\rangle|^2}$$

$$\leq \frac{1}{2} + \frac{1}{2}\sqrt{(p_\theta+q_\theta)^2 - (p_\theta-q_\theta)^2\sin^2(\theta+\alpha)} \tag{33}$$

We know that $S = p_\theta + q_\theta$ is a constant, independent of θ. From Eq. (25) and the fact that $F'(\rho) = \frac{1}{2}$, we have that $2\sqrt{p_\theta q_\theta} = 1 - S$. We can easily infer:

$$(p_\theta - q_\theta)^2 = S^2 - 4p_\theta q_\theta = 2S - 1 \tag{34}$$

Eq. (33) then becomes:

$$\Pr[\text{guess } Y_H|\theta] \leq \frac{1}{2} + \frac{1}{2}\sqrt{S^2 - (2S-1)\sin^2(\theta + \alpha)} \tag{35}$$

It also holds that $F'(\rho) \leq S$ which implies $S \geq \frac{1}{2}$. When $S \in [1/2, 1]$, we can analytically show that $P[\rho|\theta\text{-protocol}]$ is maximal for $S = 1$. This gives

$$P[\rho|\theta\text{-protocol}] \leq \frac{1}{\pi}\int_0^\pi \frac{1}{2} + \frac{1}{2}\sqrt{1 - \sin^2(\theta + \alpha)}d\theta = \frac{1}{2} + \frac{1}{\pi} \approx 0.818. \tag{36}$$

Now if the parties are running the XY-protocol, then instead of integrating from 0 to π, we just need to add the cases where $\theta = 0$ and $\theta = \pi/2$. We have:

$$\begin{aligned} P[\rho|XY\text{-protocol}] &= \frac{1}{2}\big[\Pr[\text{guess } Y_H|0] + \Pr[\text{guess } Y_H|\frac{\pi}{2}]\big] && (37) \\ &\leq \frac{1}{2} + \frac{1}{4}\big[\cos^2(\alpha) - \sin^2(\alpha)\big] && (38) \end{aligned}$$

Since α is a characteristic of the state, and can therefore be chosen by the source, the above probability is maximized for $\alpha = -\pi/4$, and is equal to $\cos^2(\pi/8) \approx 0.854$.

□

Loss

If the Verifier is willing to accept an individual loss rate λ, then a cheating party can profit from declaring 'loss' in order to increase the probability of passing the test. We are interested to see how the two protocols behave in the presence of loss. We concentrate on checking for genuine multipartite entanglement. Since the dishonest parties have full control of the source, and in particular their part (including purification), we treat the dishonest parties as a single system. That is, we say that a source state $|\psi\rangle_{H,D}$ is genuinely multipartite entangled if it is entangled across all bipartite cuts where D is treated as a single party (that is all D systems are on one side of the bipartition).

Looking only at GME in this way greatly simplifies the analysis. We now wish to bound the probability of passing the test for states which are not GME, that is, there exists a partition such that $|\psi\rangle_{H,D}$ is separable. To bound this take all the honest players which are on D's side of this partition, and imagine the dishonest party has control of them too, *i.e.* we have a bigger D including these (this cannot

but help the dishonest party pass the test). We thus concentrate on product states of the form $|H\rangle_H \otimes |D\rangle_D$.

The θ-protocol. Let $|H\rangle = \alpha|0\rangle^{\otimes k} + e^{i\theta'}\beta|1\rangle^{\otimes k} + \gamma|\mathcal{X}\rangle$ the state shared by the honest players with $|\mathcal{X}\rangle$ orthogonal to both $|0\rangle^{\otimes k}$ and $|1\rangle^{\otimes k}$ and $\alpha, \beta \in \mathbb{R}^+$. For a fixed θ and using the characterization of our test, the honest players will output $Y_H = 0$ with probability

$$\Pr[Y_H = 0|\theta] = \frac{|\gamma|^2}{2} + |\frac{\alpha}{\sqrt{2}} + \frac{\beta}{\sqrt{2}}e^{i(\theta'-\theta)}|^2 = \frac{1}{2} + \alpha\beta\cos(\theta' - \theta) \tag{39}$$

The dishonest parties want to guess Y_H. They will guess $Y_H = 0$ when $\cos(\theta'-\theta) \geq 0$ and $Y_H = 1$ otherwise, and they will succeed with probability $\frac{1}{2} + \alpha\beta|\cos(\theta' - \theta)|$. This probability is maximized for $\alpha, \beta = \frac{1}{\sqrt{2}}$. Without any loss, the dishonest players succeed with probability:

$$\frac{1}{\pi}\left(\int_0^{\pi} \frac{1}{2} + \frac{1}{2}|\cos(\theta' - \theta)|d\theta\right) = \frac{2}{\pi}\int_{\theta'}^{\theta'+\pi/2} \cos^2(\frac{\theta}{2})d\theta \tag{40}$$

In the case where there is loss, the cheating players can post-select on a λ fraction of the angles. This is the only thing they can do since their state $|D\rangle$ is unentangled with $|H\rangle$. The worst angles are the ones close to $\pi/2 + \theta'$. In that case, when the state is tested, the cheating players pass the test with probability:

$$P(\lambda) = \frac{2}{\pi(1-\lambda)}\int_{\theta'}^{\theta'+\pi(1-\lambda)/2} \cos^2(\frac{\theta}{2})d\theta = \frac{2}{\pi(1-\lambda)}\int_0^{\pi(1-\lambda)/2} \cos^2(\frac{\theta}{2})d\theta \tag{41}$$

The XY-protocol. Analyzing this protocol is done in a similar way as before. We start from $|H\rangle = \alpha|0\rangle^{\otimes k} + e^{i\theta'}\beta|1\rangle^{\otimes k} + \gamma|\mathcal{X}\rangle$ with $\alpha, \beta \in \mathbb{R}^+$.

- The honest parties receive an even number of Pauli Y measurement requests: this corresponds to them performing a θ-test with $\theta = 0$. This means that the honest players output $Y_H = 0$ with probability $\frac{1}{2} + \alpha\beta\cos(\theta')$. The optimal dishonest strategy is to guess $Y_H = 0$ when $\cos(\theta') \geq 0$. Otherwise, they guess $Y_H = 1$. This overall strategy will succeed with probability $\frac{1}{2} + \alpha\beta|\cos(\theta')|$. Notice that this is maximized for $\alpha, \beta = \frac{1}{\sqrt{2}}$ which gives $\Pr[\text{pass test}|\text{even } Y] = \frac{1}{2} + \frac{|\cos(\theta')|}{2}$.
- The honest parties receive an odd number of Pauli Y measurement requests: this corresponds to them performing a θ-test with $\theta = \pi/2$. Similarly as above, we can show that $\Pr[\text{pass test}|\text{odd } Y] = \frac{1}{2} + \frac{|\cos(\theta'+\pi/2)|}{2} = \frac{1}{2} + \frac{|\sin(\theta')|}{2}$.

In the case when there is no loss, the pass probability of state $|H\rangle$ is maximised for $\theta' = \pi/4$, since for both measurement settings of the honest parties, the pass probability is $cos^2(\pi/8) \approx 0.854$. For 50% loss, the pass probability of state $|H\rangle$ is maximised for $\theta' = 0$, since whenever the dishonest party is asked to measure in

the Pauli Y basis, he declares loss, resulting in a pass probability equal to 1. For any amount of loss between these two values, the optimal dishonest strategy is a probabilistic mixture of the two pure strategies:

- With probability 2λ the source sets $\theta' = 0$, and whenever the dishonest party receives Y, he declares loss.
- With probability $1-2\lambda$ the source sets $\theta' = \pi/4$.

Let $Q(\lambda)$ be the probability that the dishonest parties pass the test, conditioned on not declaring loss. We have:

$$Q(\lambda) = \frac{1}{1-\lambda}\left(\lambda \cdot \Pr[\text{pass test}|\theta' = 0, X] + (1-2\lambda)\cdot \Pr[\text{pass test}|\theta' = \pi/4]\right) \quad (42)$$

$$= \frac{\lambda + (1-2\lambda)\cdot 0.854}{1-\lambda} \quad (43)$$

Supplementary Figure 1 shows the difference in the pass probability for the two tests when the amount of tolerated loss increases. Here, $Q(\lambda)$ is plotted for the XY-protocol test and $P(\lambda)$ is plotted for the θ-protocol test.

Supplementary Note 2

State generation

The generation of photon pairs in our setup is achieved by spontaneous four-wave mixing (SFWM) in fiber sources exploiting birefringent phase-matching (1, 2). The fibers are strongly birefringent ($\Delta n = 4 \times 10^{-4}$) and microstructured, with the phase-matching generating signal-idler pairs cross-polarization to the pump laser. The waveguide contributions to the dispersion in addition to the birefringence tailor the SFWM to the generation of naturally narrowband spectrally uncorrelated photons when pumped with Ti-Sapphire laser pulses at 726nm. This is achieved at the flat region of the phase-matching curves upon which the idler photons ($\lambda_i = 871$ nm) are group velocity matched to the pump pulse so that they become spectrally broad ($\Delta\lambda_i = 2.2$ nm) whilst the signal photons ($\lambda_s = 623$ nm) are intrinsically narrowband ($\Delta\lambda_s = 0.3$ nm). This narrowband phase-matching results in a Joint-Spectral Amplitude (JSA) which is highly separable for a wide range of pump bandwidths and thus single photons of high purity can be produced. The pump bandwidth can then be tuned to minimize the effects of deviating from the flat region at 726 nm whilst reducing the self-phase modulation caused by short pulses, to arrive at an optimal pump bandwidth of $\Delta\lambda_p = 1.7$ nm.

The fiber sources are then positioned in Sagnac-loop configurations in which the pump pulse is set to diagonal polarization and split at a polarizing beam-splitter

(PBS), after which it is launched into the fiber in both directions simultaneously. The 90° rotation of the fiber axis between its two facets results in the pump light being strongly suppressed out of the port of the PBS it entered, whilst the generated signal-idler pairs from each facet of the fiber are cross-polarized to the pump that generated them, so exit from the other port to the pump. The pairs generated from each direction traverse the same mode in reverse so on exiting the PBS they coherently share the same spatio-temporal mode and create the state $\frac{1}{\sqrt{2}}(|H\rangle_s |H\rangle_i + e^{i\theta} |V\rangle_s |V\rangle_i)$ up to some phase θ.

The generation of three- and four-photon GHZ states in our setup is then achieved by a parity check, or 'fusion', with post-selection (3–6). Fusion processes of this sort require photons originating from two distinct sources to be indistinguishable in all degrees of freedom, however the fabrication of microstructured fibres can result in small inhomogeneities between fiber samples. To overcome these inhomogeneities one fiber source is temperature tuned so that the spectra of the signal photons match the spectra of the signal photons in the other fiber. This reduces the distinguishability. The spectra of the signal photons from each source were measured for a range of pump bandwidths and Gaussians fitted to determine their central wavelength. The second source was then temperature tuned using a Peltier cooler to $23.7°C$ (relative to the ambient $17.6°C$) to achieve optimal indistinguishability in the spectra (see Supplementary Figure 2). It is useful to note here that despite the inhomogeneous distribution of heat to the fiber, which results in significant broadening of the idler photon, the narrowband phase-matching scheme ensures that the signal photon remains narrowband. However, note there are still small differences between the signal spectra that arise from inhomogeneities in the fibre and these reduce the maximum fidelity achievable.

The generation of the four-photon GHZ state proceeds by overlapping the signal photons from two Bell pair sources at a PBS and post-selecting the event in which one photon is detected at each output port. On the other hand, the three-photon GHZ state requires one of the sources to contribute just a single heralded signal photon in the state $|D\rangle = \frac{1}{\sqrt{2}}(|H\rangle + |V\rangle)$ and post-selecting similarly. This is achieved by pumping the second source in only one direction and rotating the heralded signal photon with a half-wave plate.

Arbitrary local projective measurements are achieved by polarisation rotations using pairs of half- and quarter-wave plates, followed by polarising beam splitters (PBSs) to spatially separate the two eigenstates of polarisation, before collection into 8 silicon avalanche photodiode detectors. Pairs of automated achromatic half- and quarter-wave plates were calibrated to account for the chromatic deviations at signal and idler wavelengths, and numerical methods were used to find wave plate angles to map the input states to the states closest to the ideal projection vectors.

Note that due to the chromatic deviations of wave plates, not all rotations can necessarily be achieved, so to allow the Pauli bases and the X-Y equator to be reached, appropriate approximate states were chosen by fiber polarizers for input to the measurement stage.

Higher-order terms from sources

4-qubit GHZ

The state generated by four-wave mixing in one source in an 'entangled configuration' can be written as (7)

$$\begin{aligned} |\psi\rangle_{s,i} &= \mathcal{N}(|0,0\rangle_{s,i} + \alpha(|1_H,1_H\rangle_{s,i} + |1_V,1_V\rangle_{s,i}) \\ &\quad + \alpha^2(|2_H,2_H\rangle_{s,i} + |2_V,2_V\rangle_{s,i} + |1_H1_V,1_H1_V\rangle_{s,i}) + \mathcal{O}(\alpha^3)), \end{aligned} \tag{44}$$

where $\mathcal{N}$ is a normalisation constant, $|\alpha|^2 = \bar{n}/(\bar{n}+1)$ is the mean number of signal-idler pairs generated in a pulse and $\left|\ell_{H/V}\right\rangle_k = \frac{1}{\sqrt{\ell!}}(\hat{a}^\dagger_{H/V,k})^\ell\,|0\rangle_k$ for mode k. Taking two sources in the entangled configuration we have the starting state

$$\begin{aligned} |\psi\rangle_{s_1,i_1,s_2,i_2} &= \mathcal{N}(|0,0\rangle_{s_1,i_1} + \alpha(|1_H,1_H\rangle_{s_1,i_1} + |1_V,1_V\rangle_{s_1,i_1}) \\ &\quad + \alpha^2(|2_H,2_H\rangle_{s_1,i_1} + |2_V,2_V\rangle_{s_1,i_1} + |1_H1_V,1_H1_V\rangle_{s_1,i_1}) + \mathcal{O}(\alpha^3)) \otimes \\ &\quad (|0,0\rangle_{s_2,i_2} + \alpha(|1_H,1_H\rangle_{s_2,i_2} + |1_V,1_V\rangle_{s_2,i_2}) \\ &\quad + \alpha^2(|2_H,2_H\rangle_{s_2,i_2} + |2_V,2_V\rangle_{s_2,i_2} + |1_H1_V,1_H1_V\rangle_{s_2,i_2}) + \mathcal{O}(\alpha^3)), \end{aligned} \tag{45}$$

which gives 35 terms when expanded up to α^3. Applying the PBS transformations for the fusion: $\hat{a}_{H,s_1} \to \hat{a}_{H,s_1}$,$\hat{a}_{V,s_1} \to \hat{a}_{V,s_2}$, $\hat{a}_{H,s_2} \to \hat{a}_{H,s_2}$ and $\hat{a}_{V,s_2} \to \hat{a}_{V,s_1}$, and taking terms that have at least one photon in each mode we have the state

$$\begin{aligned} |\psi\rangle &= \mathcal{N}(\alpha^2(|1_H,1_H,1_H,1_H\rangle + |1_V,1_V,1_V,1_V\rangle) + \alpha^3(|2_H,2_H,1_H,1_H\rangle \\ &\quad + |1_H,1_H,2_H,2_H\rangle + |2_V,1_V,1_V,2_V\rangle + |1_V,2_V,2_V,1_V\rangle + \frac{1}{2}(|1_H1_V,1_H,1_V,1_H1_V\rangle \\ &\quad + |1_H1_V,1_H1_V,1_V,1_V\rangle + |1_H,1_H1_V,1_H1_V,1_H\rangle + |1_V,1_V,1_H1_V,1_H1_V\rangle)))_{s_1,i_1,s_2,i_2}, \end{aligned} \tag{46}$$

where the terms with α^2 lead to the desired GHZ state and higher-order terms with α^3 cause the state to be non-ideal. Here we have not included the possibility of further postselection depending on the measurement basis. For example, in the H/V basis the last 4 terms can be dropped, as two photons in a single mode will lead to both detectors from the polarisation analysis of that mode giving a click. This is not the case for all bases however.

The fidelity of $|\psi\rangle$ with respect to the ideal GHZ state is $F = 2\alpha^4/(2\alpha^4 + 5\alpha^6)$. For the pump power used in our experiment of $P = 7$ mW in each fibre in each direction we have $\bar{n} = 0.05$ and therefore $\alpha = 0.22$, leading to a fidelity of $F = 0.89$. Thus, higher-order emissions up to α^3 reduce the quality of the state, as measured

using the fidelilty, by 11%. Terms with α^4 are 0.22 times smaller than those with α^3 and will therefore have a contribution of only $1-2\%$. At the pump power used we have a rate of four-folds of 1-2 s^{-1}. An interesting question is whether the higher-order emissions can be used by the dishonest parties to gain an advantage when loss is present. This is a system dependent issue which we leave for future work. However, we note that regardless of this, by using a smaller pump power one can reduce the impact of higher order terms on the fidelity in our setup, although at the expense of the overall four-fold rate. For example, with $P = 1$ mW one can reduce the impact on the fidelity to only 2%.

3-qubit GHZ

The state generated by four-wave mixing in one source in a 'product configuration' can be written as (7)

$$|\psi\rangle_{s,i} = \mathcal{N}(|0,0\rangle_{s,i} + \alpha\,|1_H,1_H\rangle_{s,i} + \alpha^2\,|2_H,2_H\rangle_{s,i} + \mathcal{O}(\alpha^3)). \quad (47)$$

Taking one source in the product configuration and the other in the entangled configuration we have the starting state

$$\begin{aligned}|\psi\rangle_{s_1,i_1,s_2,i_2} = {} & \mathcal{N}(|0,0\rangle_{s_1,i_1} + \alpha\,|1_H,1_H\rangle_{s_1,i_1} + \alpha^2\,|2_H,2_H\rangle_{s_1,i_1} + \mathcal{O}(\alpha^3)) \otimes \\ & (|0,0\rangle_{s_2,i_2} + \alpha(|1_H,1_H\rangle_{s_2,i_2} + |1_V,1_V\rangle_{s_2,i_2}) \\ & +\alpha^2(|2_H,2_H\rangle_{s_2,i_2} + |2_V,2_V\rangle_{s_2,i_2} + |1_H1_V,1_H1_V\rangle_{s_2,i_2}) + \mathcal{O}(\alpha^3)).\end{aligned} \quad (48)$$

which gives 20 terms when expanded up to α^3. Applying the HWP on mode s_1: $\hat{a}_{H,s_1} \to \frac{1}{\sqrt{2}}(\hat{a}_{H,s_1} + \hat{a}_{V,s_1})$, and the PBS transformations for the fusion: $\hat{a}_{H,s_1} \to \hat{a}_{H,s_1}$, $\hat{a}_{V,s_1} \to \hat{a}_{V,s_2}$, $\hat{a}_{H,s_2} \to \hat{a}_{H,s_2}$ and $\hat{a}_{V,s_2} \to \hat{a}_{V,s_1}$, and taking terms that have at least one photon in each mode we have the state (conditioned on a detection of one or more photons in mode i_1)

$$\begin{aligned}|\psi\rangle = {} & \mathcal{N}[\frac{\alpha^2}{\sqrt{2}}(|1_H,1_H,1_H\rangle + |1_V,1_V,1_V\rangle) + \frac{\alpha^3}{\sqrt{2}}\big(|1_H,2_H,2_H\rangle + |2_V,1_V,2_V\rangle \\ & +\frac{1}{2}\,|1_H1_V,1_H,1_H1_V\rangle + \frac{1}{2}\,|1_V,1_H1_V,1_H1_V\rangle + \frac{1}{\sqrt{2}}\,|2_H,1_H,1_H\rangle + \frac{1}{\sqrt{2}}\,|1_V,2_V,1_V\rangle \\ & + |1_H1_V,1_V,1_V\rangle + |1_H,1_H1_V,1_H\rangle\big)]_{s_1,s_2,i_2},\end{aligned} \quad (49)$$

where the terms with α^2 lead to the desired GHZ state and higher-order terms with α^3 cause the state to be non-ideal. Here we have again not included the possibility of further postselection depending on the measurement basis.

The fidelity of $|\psi\rangle$ with respect to the ideal GHZ state is $F = \alpha^4/(\alpha^4 + \frac{11}{4}\alpha^6)$. For the pump power used in our experiment of $P = 7$ mW in each fibre in each direction (with the source in the product configuration only pumped in one direction) we have $\bar{n} = 0.05$ and therfore $\alpha = 0.22$, leading to a fidelity of $F = 0.88$. Thus, higher-order

emissions up to α^3 reduce the quality of the state, as measured using the fidelity, by 12%. Terms with α^4 are 0.22 times smaller than those with α^3 and will therefore have a contribution of only $1 - 2\%$. Again, for a low pump power of $P = 1$ mW one can reduce the impact of the higher order terms on the fidelity to 2%.

Supplementary References

1. M. Halder, J. Fulconis, B. Cemlyn, A. Clark, C. Xiong, W. J. Wadsworth and J. G. Rarity, Nonclassical 2-photon interference with separate intrinsically narrowband fibre sources, Optics Express **17**, 4670-4676 (2009).

2. A. Clark, B. Bell, J. Fulconis, M. M. Halder, B. Cemlyn, O. Alibart, C. Xiong, W. J. Wadsworth and J. G Rarity, Intrinsically narrowband pair photon generation in microstructured fibres, New J. Phys. **13**, 065009 (2011).

3. T. B. Pittman, B. C. Jacobs and J. D. Franson, Probabilistic quantum logic operations using polarizing beam splitters, Phys. Rev. A **64**, 062311 (2001).

4. J.-W. Pan, C. Simon, C. Brukner and A. Zeilinger, Entanglement purification for quantum communication, Nature **410**, 1067 (2001).

5. J.-W. Pan, S. Gasparoni, R. Ursin, G. Weihs and A. Zeilinger, Experimental entanglement purification of arbitrary unknown states, Nature **423**, 417 (2003).

6. B. Bell, A. Clark, M. S. Tame, M. Halder, J. Fulconis, W. Wadsworth and J. Rarity, Experimental characterization of photonic fusion using fiber sources, New J. Phys. **14**, 023021 (2012).

7. J. Fulconis, O. Alibart, W. J. Wadsworth and J. G. Rarity, Quantum interference with photon pairs using two micro-structured fibres, New J. Phys. **9**, 276 (2007).
